# CD200 Baseline Serum Levels Predict Prognosis of Chronic Lymphocytic Leukemia

**DOI:** 10.3390/cancers13164239

**Published:** 2021-08-23

**Authors:** Giovanni D’Arena, Candida Vitale, Marta Coscia, Daniela Lamorte, Giuseppe Pietrantuono, Francesca Perutelli, Fiorella D’Auria, Teodora Statuto, Luciana Valvano, Annamaria Tomasso, Valentina Griggio, Rebecca Jones, Giovanna Mansueto, Oreste Villani, Simona D’Agostino, Vito Viglioglia, Vincenzo De Feo, Fabrizio Calapai, Carmen Mannucci, Alessandro Sgambato, Dimitar G. Efremov, Luca Laurenti

**Affiliations:** 1 Hematology, P.O.S. Luca ASL Salerno, 84078 Vallo della Lucania, Italy; 2 Department of Molecular Biotechnology and Health Sciences, University of Torino and Division of Hematology, A.O.U. Città della Salute e della Scienza di Torino, 10126 Torino, Italy; candida.vitale@unito.it (C.V.); marta.coscia@unito.it (M.C.); francesca.perutelli@unito.it (F.P.); valentina.griggio@unito.it (V.G.); rebecca.jones@unito.it (R.J.); 3 Laboratory of Pre-Clinical and Translational Research, Centro di Riferimento Oncologico della Basilicata (IRCCS CROB), 71013 Rionero in Vulture, Italy; lamortedaniela@yahoo.it; 4 Hematology and Stem Cell Transplantation Unit, Centro di Riferimento Oncologico della Basilicata (IRCCS CROB), 71013 Rionero in Vulture, Italy; ematologia45@alice.it (G.P.); giovannamansueto@hotmail.com (G.M.); orestevillani6@gmail.com (O.V.); simona.dagostino@gmail.com (S.D.); 5 Laboratory of Clinical and Advanced Diagnostics, Centro di Riferimento Oncologico della Basilicata (IRCCS CROB), 71013 Rionero in Vulture, Italy; fiorella.dauria@crob.it (F.D.); teodora.statuto@libero.it (T.S.); valvano.luciana@gmail.com (L.V.); 6 Hematology Unit, Fondazione Policlinico Gemelli, IRCCS, Catholic University of Sacred Hearth, 00168 Rome, Italy; annamariatomasso2@gmail.com (A.T.); Luca.Laurenti@unicatt.it (L.L.); 7 Scientific Direction, Centro di Riferimento Oncologico della Basilicata (IRCCS CROB), 71013 Rionero in Vulture, Italy; vito.viglioglia@crob.it (V.V.); alessandro.sgambato@crob.it (A.S.); 8 Department of Pharmacy, University of Salerno, 84084 Salerno, Italy; defeo@unisa.it; 9 Department of Chemical, Biological, Pharmaceutical and Environmental Sciences, University of Messina, 98124 Messina, Italy; fabrizio.calapai@unime.it; 10 Department of Biomedical and Dental Sciences and Morphofunctional Imaging, University of Messina, 98124 Messina, Italy; cmannucci@unime.it; 11 Molecular Hematology, International Center for Genetic Engineering and Biotechnology, 341949 Trieste, Italy; efremov@icgeb.org

**Keywords:** CD200, chronic lymphocytic leukemia, prognosis, serum

## Abstract

**Simple Summary:**

This study aimed at investigating the prognostic significance of the soluble form of CD200 antigen evaluated at diagnosis in patients with chronic lymphocytic leukemia (CLL). In a large cohort of patients, we found that more aggressive features and a worse prognosis are correlated with higher baseline serum levels of CD200. These data support the relevant role of CD200 not only as a diagnostic tool but also as a prognostic indicator and a potential therapeutic target in CLL.

**Abstract:**

Membrane-bound CD200 is overexpressed in chronic lymphocytic leukemia (CLL), and there is some evidence that its soluble ectodomain (sCD200) could also be involved in the pathophysiology and the disease. However, very little is known about sCD200’s prognostic significance. sCD200 was tested at diagnosis in 272 patients with CLL and in 78 age- and sex-matched healthy subjects using a specific human CD200 (OX-2 membrane glycoprotein) ELISA kit. A significantly higher concentration of sCD200 was found in CLL patients compared to controls. In our cohort, sCD200 was significantly higher in patients who were older than 66 years, with Binet stage C, unmutated IgVH and unfavorable (del11q or del17p) FISH. Time-to-first treatment and overall survival were significantly shorter in patients with higher sCD200 concentration, using as a cut-off 1281 pg/mL, the median value for sCD200 concentration in the whole CLL cohort. However, the prognostic impact of sCD200 was not confirmed in multivariate analysis. Baseline sCD200 values appeared to have an impact on the response to chemotherapy or chemo-immunotherapy, but not to targeted agents. Collectively, our data show that sCD200 serum levels correlate with more aggressive clinical and biological features and are able to predict a worse prognosis. This work supports the relevant role of CD200 not only as a diagnostic tool but also as a prognostic indicator and a potential therapeutic target in CLL.

## 1. Introduction

CD200 is a single-pass, type I membrane glycoprotein belonging to the immunoglobulin superfamily [1]. Thymocytes, B lymphocytes, a subset of T lymphocytes, endothelial cells, some dendritic cells, neurons, kidney glomeruli and syncytiothrophoblasts express CD200, while the expression of its receptor (CD200R) is more restricted and involves primarily myeloid leukocytes, such as macrophages, dendritic cells and mast cells, as well as B lymphocytes and a subset of T lymphocytes [2]. 

The anti-CD200 monoclonal antibody identifies a surface membrane antigen that has recently emerged as a useful tool to better discriminate among neoplasias of mature B lymphocytes [3]. In fact, CD200 is particularly helpful in distinguishing some disease entities, such as chronic lymphocytic leukemia (CLL) and mantle cell lymphoma (MCL), whose clinical behavior and prognosis is quite different [3,4,5]. For this reason, CD200 has now gained a relevant role in the monoclonal antibody panels to be tested in the diagnostic work-up of lymphoid chronic leukemias by means of flow cytometry [4]. 

It is well-established that CD200 is a molecule with a negative immunoregulatory function and is overexpressed on cells of various tumors including CLL [6,7,8]. CLL cells can influence T-cell function via expression of cell-surface molecules and soluble factors [9,10], and it has also been demonstrated that CD200 is functionally involved in modulating the CLL microenvironment, which is crucial for the survival and proliferation of CLL cells [11,12,13]. Regarding the possible prognostic role of the intensity of CD200 expression in CLL, to date only a limited number of studies have been conducted, and results have not been definitive [14]. CD200 is also emerging as a potential therapeutic target in CLL [15,16,17], and promising results have been recently obtained by a first-in-human study investigating samalizumab, a recombinant humanized monoclonal anti-CD200 antibody, suggesting that CD200 could represent a novel target for immune checkpoint inhibitor drugs [18]. 

Interestingly, CD200 is not only present as a membrane-bound molecule but also in its soluble forms (sCD200), which can be shed from the CLL cell surface by stimulation with phorbol 12-myristate 13-acetate and TLR7 agonists in vitro [12]. There is also some evidence that serum levels of sCD200 could be related to disease progression in patients with CLL [19,20,21]. Moreover, as postulated for the membrane-bound counterpart, sCD200 may exert immunoregulatory functions. Released sCD200 is able to engage CD200R1, inducing intracellular signaling, and it has been reported that sCD200 is able to promote CLL cells’ growth in immunodeficient mice, in a process that critically involves T cells [20]. Of note, a subpopulation of CD4+ T cells have previously been shown to express CD200R [22], and CD200+ CLL cells and CD200RCD4+ T cells seem to colocalize in the tumor microenvironment. 

With the aim of verifying whether sCD200 tested at diagnosis in CLL patients is correlated with clinical and biological features of the disease and is able to predict the prognosis, we performed the present study on a large cohort of CLL patients consecutively seen at our institutions.

## 2. Materials and Methods

Patients with CLL or small lymphocytic lymphoma (SLL), consecutively diagnosed at our institutions (Turin University; Fondazione Gemelli, University of Rome; Cancer Referral Center of Basilicata, Rionero in Vulture), and for whom stored serum samples collected at diagnosis were available, were included in the study. We analyzed sera from 272 patients and from 78 age- and sex-matched healthy donors (HD), used as normal controls. For 12 patients with CLL/SLL, a post-treatment sample was also available. CLL/SLL diagnosis and post-treatment response evaluation were performed according to iwCLL-NCI criteria [23]. Clinical data were collected from electronic medical records at each Institution. 

This retrospective study was performed according to the informed consent procedure approved by the local internal review board (Protocol no. 20140040750—18.11.2014), and it conforms to the provisions of the Declaration of Helsinki.

The concentration of CD200 (OX-2 membrane glycoprotein) in human serum was detected by enzyme-linked immunosorbent assay technology (ELISA) manufactured by Wuhan Fine Biotech Co., Ltd. (Wuhan, Hubei, China) (Catalogue number: EH0077). The analysis was carried out by Labospace S.r.l. (Via Virgilio Ranzato, 12, 20128 Milano, Italy) following the procedure described in the kit manual. All the serum samples were diluted with the buffer solution with the proper dilution factor and were analyzed in duplicate. The ELISA technology was employed by the following assay principle. The captured antibody (mouse monoclonal) was pre-coated onto 96-well plates, and the biotin-conjugated rabbit polyclonal antibody was used for detection. The standards, the test samples and the biotin-conjugated detection antibody were added to the wells subsequently and washed with the wash buffer. Horseradish peroxidase (HRP)–streptavidin complex was added, and unbound conjugates were washed away with wash buffer. The 3.3′, 5.5′-tetramethylbenzidine (TMB) substrates were used to detect HRP enzymatic reaction. The reaction between the TMB substrate and the HRP led to a blue-colored reaction product. Then, the reaction was quenched by the addition of the acidic stop solution, obtaining a yellow final product. The intensity of the yellow color was proportional to the CD200 amount captured on the plate. The O.D. absorbance was registered, in correspondence with the typical wavelength of 450 nm, by using a microplate reader (Victor 3V Mod. 1420 S.N. 4206516, Perkin Elmer, Shelton, CT, USA). The concentration value (pg/mL) of the CD200 was calculated by the Software WorkOut 2.5.

Descriptive statistics were used to summarize patients’ characteristics. Patient groups were compared using the Mann–Whitney test. The correlation between variables was assessed by the nonparametric Spearman’s rank correlation test. The difference between pre- and post-treatment sCD200 value was assessed with the Wilcoxon matched-pairs signed rank test. Time-to-first treatment (TTFT) was defined as the time interval between the date of CLL diagnosis and the date of first treatment or last follow-up. Overall survival (OS) was defined as time from diagnosis to death for any cause or last follow-up. TTFT and OS were estimated using the Kaplan–Meier method, and differences between groups were evaluated with the log-rank test. Multivariate Cox proportional hazards regression models were fitted to assess associations between patients’ characteristics and time-dependent variables. For the multivariate analysis, covariates were selected based on the significance in univariate analysis. Statistical analyses were performed using the IBM SPSS Statistics software version 22.0 for Windows (IBM Corp., Armonk, NY, USA). A *p* value < 0.05 was considered significant.

## 3. Results

Clinical and biological features of patients at study entry are summarized in Table 1. For the entire cohort, median follow-up was 106 months. Median TTFT and OS were 74 months and 299 months, respectively.

**Figure 1:**
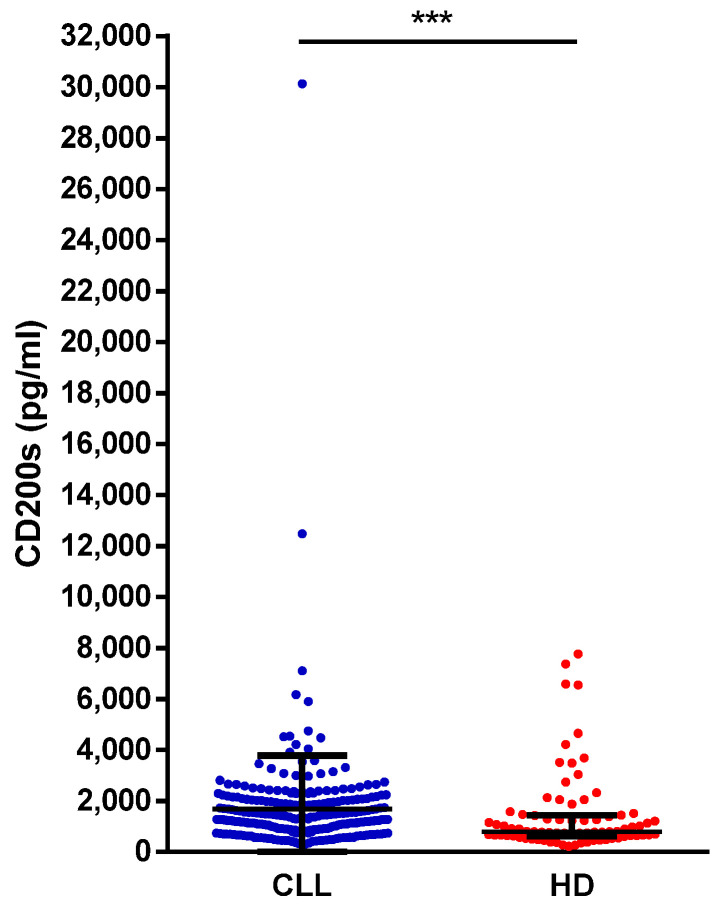
sCD200 in patients with CLL and in HD. Each dot represents the value for the sCD200 serum concentration from a single patient sample. Lines represent median values and interquartile ranges. *** *p* < 0.001.

The HD and CLL cohorts were balanced in terms of age and sex distribution (HD, median age 63 years, range 42–100, 58% males; CLL, median age 66 years, range 33–90, 58% males). A significantly higher concentration of sCD200 was found in CLL patients compared to HD (median, 1281 pg/mL vs. 799 pg/mL; *p* = 0.0002) (Figure 1). 

In our cohort, sCD200 was significantly higher in patients ≥66 vs. <66 years old (median sCD200, 1560 pg/mL vs. 1193 pg/mL; *p* = 0.0001), in those with Binet stage C vs. A/B (2055 pg/mL vs. 1274 pg/mL; *p* = 0.0045), in those with unmutated vs. mutated IgVH (1601 pg/mL vs. 1131 pg/mL; *p* < 0.0001), and in those with unfavorable (del11q or del17p) vs. favorable (normal or del13q or tris12) FISH (1897 pg/mL vs. 1239 pg/mL; *p* = 0.0077). On the contrary, gender, bulky disease, whole blood cell or lymphocyte count, β_2_-microglobulin serum levels and presence of autoimmune complications did not significantly correlate with sCD200 levels. 

**Figure 2:**
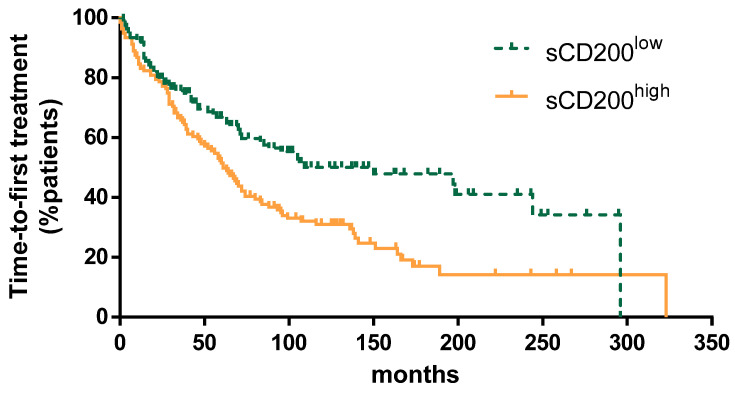
Time-to-first treatment for patients with sCD200 ^low^ vs. sCD200 ^high^.

**Figure 3:**
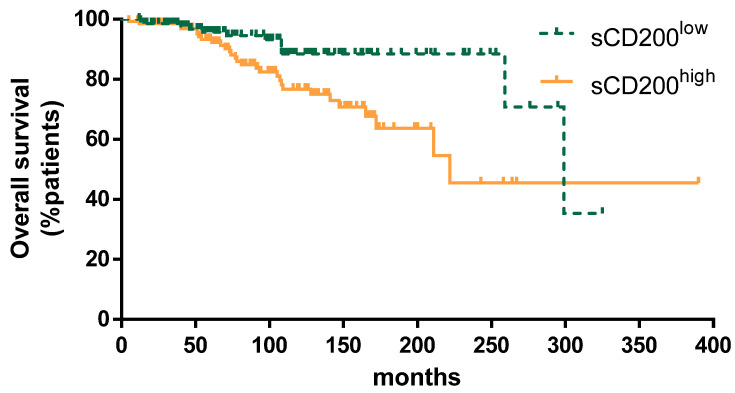
Overall survival for patients with sCD200 ^low^ vs. sCD200 ^high^.

We selected the median value for sCD200 serum concentration in the whole CLL cohort—1281 pg/mL—as the cutoff to discriminate patients with low and high sCD200. TTFT was shorter in patients with high sCD200 concentration (median TTFT, 61 vs. 150 months; *p* = 0.0006) (Figure 2). Finally, as shown in Figure 3, when patients were grouped according to sCD200 ≥ 1281 pg/mL or <1281 pg/mL, an impact on OS was also shown (median OS, 222 vs. 299 months; *p* = 0.0044). 

Time-to-first treatment was significantly longer in patients with low sCD200 compared to high sCD200 (median TTFT 150 vs. 61 months, *p* = 0.0006).

Overall survival was significantly longer in patients with low sCD200 compared to high sCD200 (median OS 299 vs. 222 months, *p* = 0.0044).

A total of 152 patients in our cohort received CLL-directed treatment (chemotherapy or chemo-immunotherapy, *n* = 123; anti-CD20 monoclonal antibody alone, *n* = 3; targeted agent, *n* = 26, among which ibrutinib ± anti-CD20 monoclonal antibody, *n* = 18; venetoclax + anti-CD20 monoclonal antibody, *n* = 4; rituximab and idelalisib, *n* = 3; zanubrutinib, *n* = 1) and had response data available. Overall, 42 patients were categorized as a CR (27%), 77 as a PR (51%), 6 as a nPR (4%) and 27 did not respond to therapy (no response, NR; 18%). Baseline sCD200 values appeared to have an impact on response to therapy (median sCD200 in CR vs. PR/NR patients, 1308 pg/mL vs. 1590 pg/mL; *p* 0.0468), and this difference seemed to increase when the analysis was restricted only to patients who had received chemotherapy or chemo-immunotherapy (1244 pg/mL vs. 1602 pg/mL; *p* = 0.0193). On the contrary, we did not find any association between baseline sCD200 values and response to targeted agents. 

**Figure 4:**
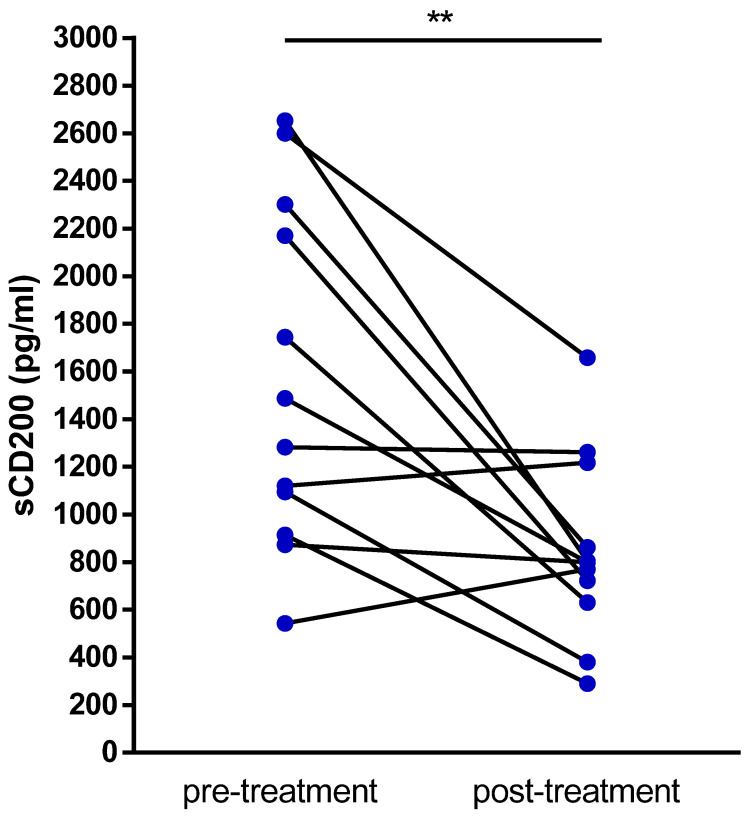
Pre- and post-treatment sCD200. Each dot represents the value for the sCD200 serum concentration from a single patient, assessed before and after chemo-immunotherapy treatment. ** *p* < 0.01.

Of interest, we evaluated sCD200 serum concentration in 12 patients before and after frontline therapy (FCR, *n* = 9; FC, *n* = 1, BR, *n* = 2). A significant decrease in sCD200 after therapy was observed (*p* = 0.0093). Specifically, 8 out of 12 patients (4 CR, 1 nPR, 3 PR) had a sCD200 decrease, while in the remaining 4 patients, sCD200 concentration remained unchanged or slightly increased (1 CR, 2 PR, 1 SD) (Figure 4, Table 2).

In a multivariate model considering only variables that were deemed significant in univariate analysis, Binet stage C, unmutated IgVH, unfavorable FISH and high sCD200 maintained their significant impact on TTFT. However, when predictors for OS were evaluated, high sCD200 was not significant (Table 3).

## 4. Discussion

CD200 is overexpressed on the surface of neoplastic cells from patients with CLL and other malignancies, delivering immunoregulatory functions [15]. A soluble form of CD200 has also been identified [11], which is shed from the CLL cell surface via proteolytic cleavage [14]. In this process, called ectodomain shedding, a cell surface protein is cleaved near its transmembrane domain, releasing biologically active soluble ectodomains [13]. 

Wong and colleagues previously evaluated the prognostic impact of sCD200 on a cohort of 82 patients with CLL, demonstrating that levels of sCD200 were greater in CLL patients as compared with healthy controls [16]. In addition, in their cohort, higher sCD200 levels correlated with tumor burden, advanced stages of the disease (Rai III and IV), more courses of treatment received (using the requirement for multiple treatment as a surrogate for worst prognosis) and higher serum β_2_-microglobulin levels. Of interest, patients with normal karyotype or del13q were treated more often if they also had high plasma levels of sCD200 [20]. 

In our larger cohort of patients, we confirmed that CLL patients have significantly higher levels of sCD200 in serum than normal subjects. Furthermore, an association between higher sCD200 levels and poor clinical and biological prognostic factors (older age, more advanced clinical stage, unmutated IgVH and unfavorable cytogenetics abnormalities) was identified. Unfortunately, the absence of data regarding TP53 mutation status prevented us from drawing conclusions regarding a possible correlation between sCD200 and this unfavorable molecular characteristic. 

It is quite surprising that bulky disease or lymphocytosis were not associated with higher levels of sCD200 in our study. The discrepancies between the findings from our series and Wong’s may be attributable to the retrospective nature of the studies and to the differences in terms of patients’ characteristics between the two cohorts. The mean age of patients in the paper by Wong and colleagues was 61 years, slightly inferior to that observed in our cohort, which was 66 years. Notably, both cohorts are constituted by younger patients, with the mean age of patients diagnosed with CLL in Western countries being 71 years, probably representing a selected group of patients referred to University or tertiary care Centers. In our study, 72% of patients had early-stage CLL (Rai 0 or Binet A), as compared to Wong’s, in which Rai stage III/IV accounted for 64.6% of cases. Additionally, patients with a normal karyotype constituted 40% of the population in our study, but only 18.6% in Wong’s. This may be the reason of the greater number of patients with normal FISH in our cohort of patients with respect to that normally found.

Of relevance, to our knowledge, this is the first paper showing a prognostic impact of baseline sCD200 in CLL, in terms of TTFT and OS, but also in terms of the quality of the response achieved after chemotherapy or chemo-immunotherapy. Interestingly, the response to targeted therapy did not seem to be influenced by the levels of sCD200 at baseline, and this may be attributable to the small number of patients receiving targeted therapy in our study and to the different kinds of response expected with this therapeutic approach compared to standard chemo-immunotherapy. It is also conceivable that the excellent efficacy of targeted treatment could somehow overcome the negative prognostic impact of baseline elevated sCD200 levels. Of note, in our series, the majority of patients treated with targeted therapies received a BTK inhibitor-based regimen, whereas only four patients were treated with venetoclax, a compound that can induce deeper responses. A possible prognostic value of sCD200 in the setting of patients treated with targeted therapies certainly needs to be confirmed in a larger cohort of patients. 

Despite the limited number of tested patients, the observation that sCD200 decreases in parallel with the CLL tumor burden reduction after treatment is also interesting and is worth confirming based on a larger series of samples.

## 5. Conclusions

Our study supports a relevant role for CD200 not only as a diagnostic tool, when evaluated in terms of surface expression, but also as a prognostic indicator, when evaluated as a soluble factor. This outlines the role of ectodomain shedding with the release of proteins exhibiting functions similar to their cell surface counterpart. In fact, both the membrane and soluble forms of CD200 can engage the CD200 receptor, which in turn can result in increased tumor growth, by means of a negative impact on tumor immunosurveillance. 

## Figures and Tables

**Table 1:** Clinical and laboratory features of CLL patients at study entry.

No. of Patients	272
Age, median (range)	66 (33–90)
Males, no (%)	157 (58%)
Lymphocyte count (×10^9^/L), (median; Range)	10.5 (5.2–218) ^1^
Hemoglobin levels (g/dL), median (range)	13.9 (7.8–17.8) ^2^
Platelet count (×10^9^/L), median (range)	188 (12–462) ^3^
Binet stage, no (%)	
A	190 (72%) ^4^
B	56 (21%)
C	18 (7%)
LDH (UI/L), median (range)	329 (131–1222) ^5^
β_2_-microglobulin (mg/L), median (range)	1.9 (1–7.3) ^6^
IgVH unmutated, no (%)	100 (43%) ^7^
FISH abnormalities, no (%)	
negative	100 (40%) ^8^
del13q	73 (29%)
tris12	38 (15%)
del17p	24 (10%)
del11q	15 (6%)
Presence of autimmune complications, no (%)	25(12%) ^9^

^1^ Data available for 255 patients, ^2^ data available for 258 patients, ^3^ data available for 257 patients, ^4^ data available for 264 patients, ^5^ data available for 209 patients, ^6^ data available for 128 patients, ^7^ data available for 232 patients, ^8^ data available for 250 patients, ^9^ data available for 201 patients. Abbreviations: IgVH, immunoglobulin heavy chain variable region; FISH, fluorescent in situ hybridization.

**Table 2:** Clinical and laboratory features of patients tested for sCD200 before and after therapy.

UPN	Age	Sex	Clinical Stage	IgVH	FISH	Pre-Treatment sCD200 (pg/mL)	Therapy	Response	Post-Treatment sCD200 (pg/mL)
114	62	F	Binet A, Rai II	U	Neg	2302.4	FC	PR	862.33
115	63	M	Binet A, Rai 0	U	Neg	1743.73	FCR	PR	630.37
116	54	M	Binet A, Rai I	U	del13q	1120.51	FCR	SD	1217.35
117	63	M	Binet C, Rai IV	M	del17p, del11q	2171.07	FCR	PR	722.129
118	44	F	Binet B, Rai II	U	del17p, del11q, tris12	873.724	FCR	PR	799.415
119	43	M	Binet A, Rai 0	*ND*	del17p, del13q, tris12	1486.73	FCR	PR	799.604
120	55	M	Binet A, Rai 0	M	del13q	2653.87	FCR	CR	803.719
121	50	M	Binet A, Rai I	M	Neg	1282.68	FCR	PR	1260.96
233	52	F	Binet B, Rai II	U	Neg	542.829	FCR	CR	770.362
238	73	M	Binet A, Rai II	M	Neg	914.563	BR	CR	290.644
219	57	M	Binet C, Rai IV	U	del11q	2599.11	FCR	CR	1658.29
197	67	M	Binet A, Rai 0	M	Neg	1094.76	BR	CR	380.056

Abbreviations. M, male; F, female; IgVH, immunoglobulin heavy chain variable region; U, unmutated; M, mutated; *ND*, not done; FISH: fluorescence in situ hybridization; neg, negative; FCR, fludarabine, cyclophosphamide, rituximab; FC, fludarabine, cyclophosphamide; BR, bendamustine, rituximab; CR, complete response; PR, partial response; SD, stable disease.

**Table 3:** Predictors of TTFT and OS in multivariate analysis.

		HR	95% CI	*p*
TTFT	Binet stage C	2.47	1.339–4.556	0.004
	IgVH U	3.024	2.084–4.389	0.0001
	del(11q) and/or del(17p)	1.836	1.169–2.885	0.008
	sCD200 ^high^	1.474	1.024–2.121	0.037
OS	Age ≥ 66	2.935	1.326–6.494	0.008
	Binet stage C	6.146	2.345–16.199	0.0001
	IgVH U	2.543	1.231–5.251	0.012
	sCD200 ^high^	2.037	0.952–4.360	NS

## Data Availability

The data that support the findings of this study are available from the corresponding author (G.D.) upon reasonable request.
